# The immunometabolic landscape of the bone marrow microenvironment in acute myeloid leukemia

**DOI:** 10.1186/s40164-022-00332-8

**Published:** 2022-10-28

**Authors:** Binyan Xu, Ziying Zhou, Yueting Wen, Zhongwei Li, Zhongxi Huang, Yuhua Li

**Affiliations:** 1 Department of Hematology, Zhujiang Hospital, Southern Medical University, Guangzhou, 510280 Guangdong People’s Republic of China; 2 Cancer Research Institute, School of Basic Medical Sciences, Southern Medical University, Guangzhou, 510515 People’s Republic of China; 3 Guangzhou Regenerative Medicine and Health Guangdong Laboratory, Guangzhou, 510005 People’s Republic of China

## Abstract

The bone marrow microenvironment of acute myeloid leukemia (AML) consists of various cell types and signaling factors, which serve as a niche supporting leukemia cells in their invasion of the human body. However, a systematic landscape of metabolic heterogeneity and its relationship with immunity in the AML microenvironment at single-cell resolution has not yet been established. Herein, we addressed this issue by analyzing 208,543 bone marrow cells from 40 AML patients and 3 healthy donors obtained from GSE130756. We focused on the metabolic preference of AML progenitor cells and diverse immune cells, especially myeloid immune cells and T cells. Accordingly, the immune evasion mechanism of leukemia cells was proposed from the view of the allocation of energy and oxygen, providing a novel direction of treatment. Finally, we tentatively proposed potential targets for AML metabolic therapy, including ENO1, GSTP1, MT-ND4L and UQCR11. Collectively, our analysis facilitates the development of personalized therapies targeting unique immunometabolic profiles.

**Supplementary Information:**

The online version contains supplementary material available at 10.1186/s40164-022-00332-8.

To the Editor,

Acute myeloid leukemia (AML) is a heterogeneous clonal disease of hematopoietic stem/progenitor cells (HSPCs) characterized by high morbidity, recidivity and lethality [1]. There is mounting evidence that the variation of the bone marrow microenvironment contributes to the immunosuppression and therapy effect of AML patients [2]. Additionally, metabolic reprogramming and trained immunity have gradually become hot targets in the tumor microenvironment, providing a novel strategy for oncotherapy [3, 4]. Thus, the metabolic regulation of the bone marrow microenvironment in leukemia summarized by our team has been published in *Blood Reviews* [5]. However, there has been no systematic assessment of how immunity influences metabolism in the leukemia niche.

Herein, we analyzed the normal and AML microenvironments with a total of 208,543 bone marrow cells from 40 AML patients and 3 healthy donors obtained from GSE130756 (Additional file 1: Table S1), which came from a high-throughput and low-cost single-cell RNA platform – Microwell seq. The major cellular components of normal and tumor cellular milieus were detected by constructing the cell atlas according to Wu’s research. [6] (Fig. 1a and Additional file 1: Fig. S1a, b).

**Fig. 1:**
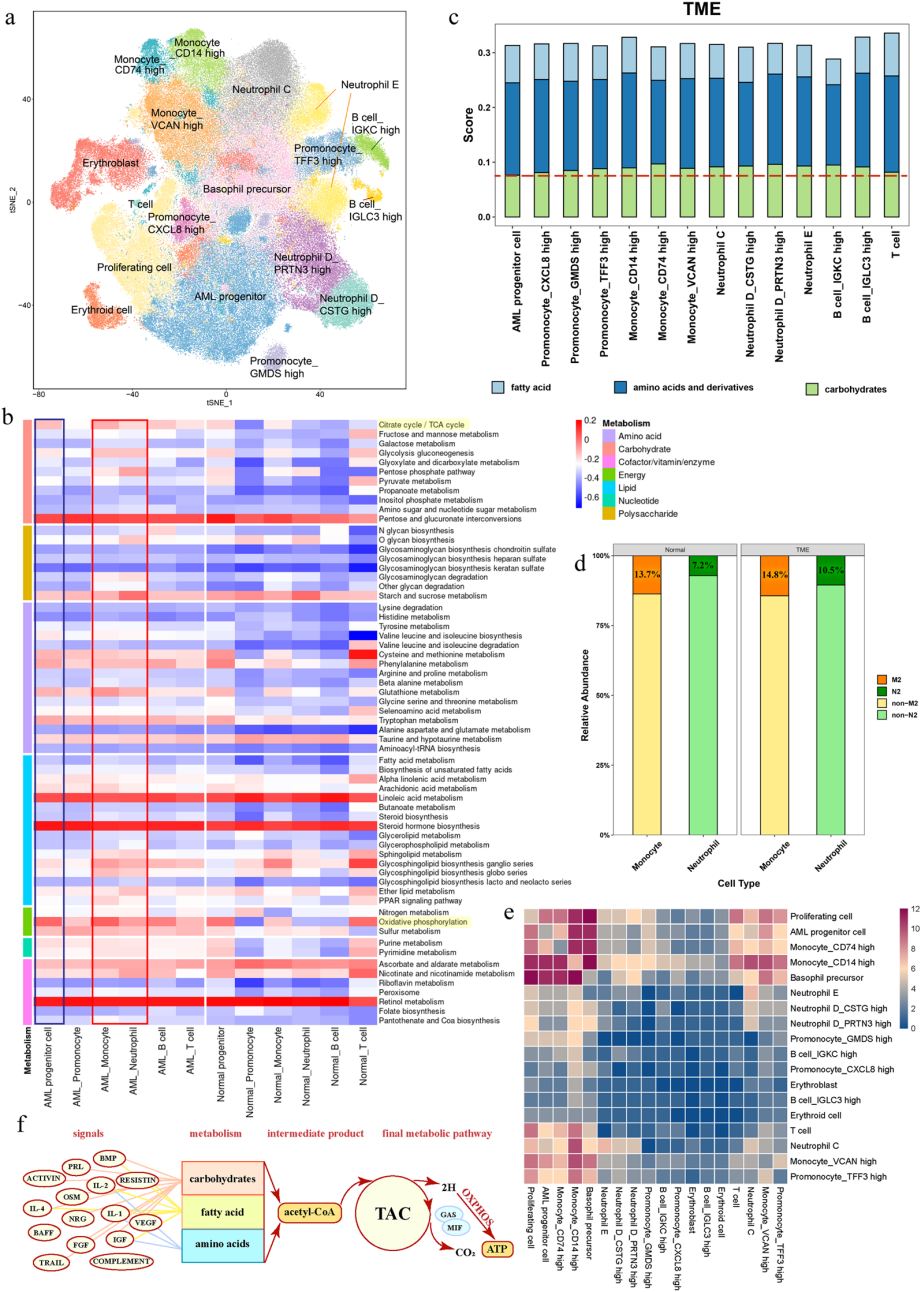
**a** Cell identification of the AML bone marrow microenvironment. **b** Metabolic heatmap of progenitor cells and immune cells from tumor and normal bone marrow microenvironments. The energy metabolism of AML progenitor cells is at a relatively low rate compared with that of tumor-infiltrating myeloid immune cells, including monocytes and neutrophils. Moreover, oxidative phosphorylation, and the tricarboxylic acid cycle play important roles in glucose metabolism in AML progenitor cells. In addition, amino acid pathways are substantially more active in tumors. Compared with the normal microenvironment, the metabolic activity of T cells in the TME decreases significantly, but myeloid immune cells increase markedly (red indicates high expression, and blue indicates low expression). **c** Metabolic preference of cells in the TME. GSVA was performed by scoring the three major metabolic pathways, and the results showed that amino acid metabolism is the main source of cell energy in the AML bone marrow microenvironment. **d** The ratios of anti-inflammatory phenotype cells (M2/N2) in normal and AML bone marrow microenvironments. The proportion of neutrophils and monocytes polarized toward the anti-inflammatory phenotype (M2/N2) in the TME is greater than that in the normal microenvironment. **e** Heatmap of cell–cell interactions. AML progenitor cells prefer to communicate with myeloid immune cells with an immunosuppressive phenotype, especially monocyte_CD14 high (red indicates high expression, and blue indicates low expression). **f** Relationships between signaling patterns and metabolism. The significant inflammatory cytokines participate in three major metabolic pathways separately

The metabolic preferences of different cells in AML and normal environments were of particular concern using GSVA. As shown in Fig. 1b, although tumor cells were characterized by rapid proliferation and metastasis [7, 8], energy metabolism was at a relatively low rate compared with that of tumor-infiltrating myeloid immune cells, which obviously increased in the AML bone marrow microenvironment. In contradiction with the Warburg effect [9], oxidative phosphorylation and the tricarboxylic acid cycle are the major energy sources of AML progenitor cells (Fig. 1b). Recent research suggests that leukemia stem cell-enriched primary populations are metabolically dormant and are more dependent on aerobic respiration than glycolysis for energy production [10], which is consistent with our results (Fig. 1b). Surprisingly, amino acid pathways are the main “building blocks” in metabolism microenvironments (Fig. 1c). Moreover, the genes related to metabolic enzymes and the metabolic activity of T cells in the tumor microenvironment (TME) decreased significantly (Additional file 1: Fig. S2a–e). By this token, the existence of AML progenitor cells promotes the formation of the TME, and most immune cells enhance their expression of metabolic genes with leukemia cells, further influencing T cells by decreasing their metabolic genes and disturbing their energy acquisition, resulting in damage to the T-cell killing effect.

It is well known that monocytes can polarize to the M1 or M2 phenotype, with anti-inflammatory or proinflammatory respectively, similar to neutrophils [11]. We successfully distinguished the phenotype of myeloid immune cells and analyzed their polarization ratio (Fig. 1d, Additional file 1: Table S2 and Fig. S3). The results show that the AML bone marrow microenvironment has high levels of myeloid cell infiltration with the increasing proportion of the anti-inflammatory phenotype. Next, we explored the nutrient and oxygen allocation of leukemia cells and tumor-infiltrating immune cells in the TME via GSEA (Additional file 1: Fig. S4–S6). Therefore, we supplemented the AML immune escape mechanism once more. Although myeloid immune cells might be the major energy consumers in the TME, rapidly proliferating leukemia cells deprive them of oxygen, and the hypoxic microenvironment then affects the direction of polarization.

Then, we depicted the communication profile by CellPhoneDB and CellChat. We found that AML progenitor cells prefer to communication with myeloid immune cells with an immunosuppressive phenotype (Fig. 1e). Moreover, the outgoing and incoming signaling profiles of each cell group were shown, and the signaling pattern in the TME was uncovered (Additional file 1: Fig. S7, S8). Combining the role played by signaling patterns in metabolism (Fig. 1f and Additional file 1: Table S3), leukemia cells affect the metabolic characteristics of immune cells through information communication with surrounding cells, and vice versa. Immune cells can further influence the leukemia under the action of signaling factors, forming a feedback loop in the TME.

Finally, a volcano map, GO terms and PPIs of metabolic differential genes related to AML progenitor cells and HSPCs are shown (Additional file 1: Fig. S9 and Fig. 2a). We screened 4 metabolic genes with statistical significance related to survival (P < 0.05) ENO1, GSTP1, MT-ND4L and UQCR11, via the GEPIA website (Fig. 2b). ENO1 is associated with glycolysis, but MT-ND4L and UQCR11 are related to oxidative phosphorylation. Moreover, GSTP1 have antitumor effects, and both serve as prognostic indicators for AML patients.

**Fig. 2:**
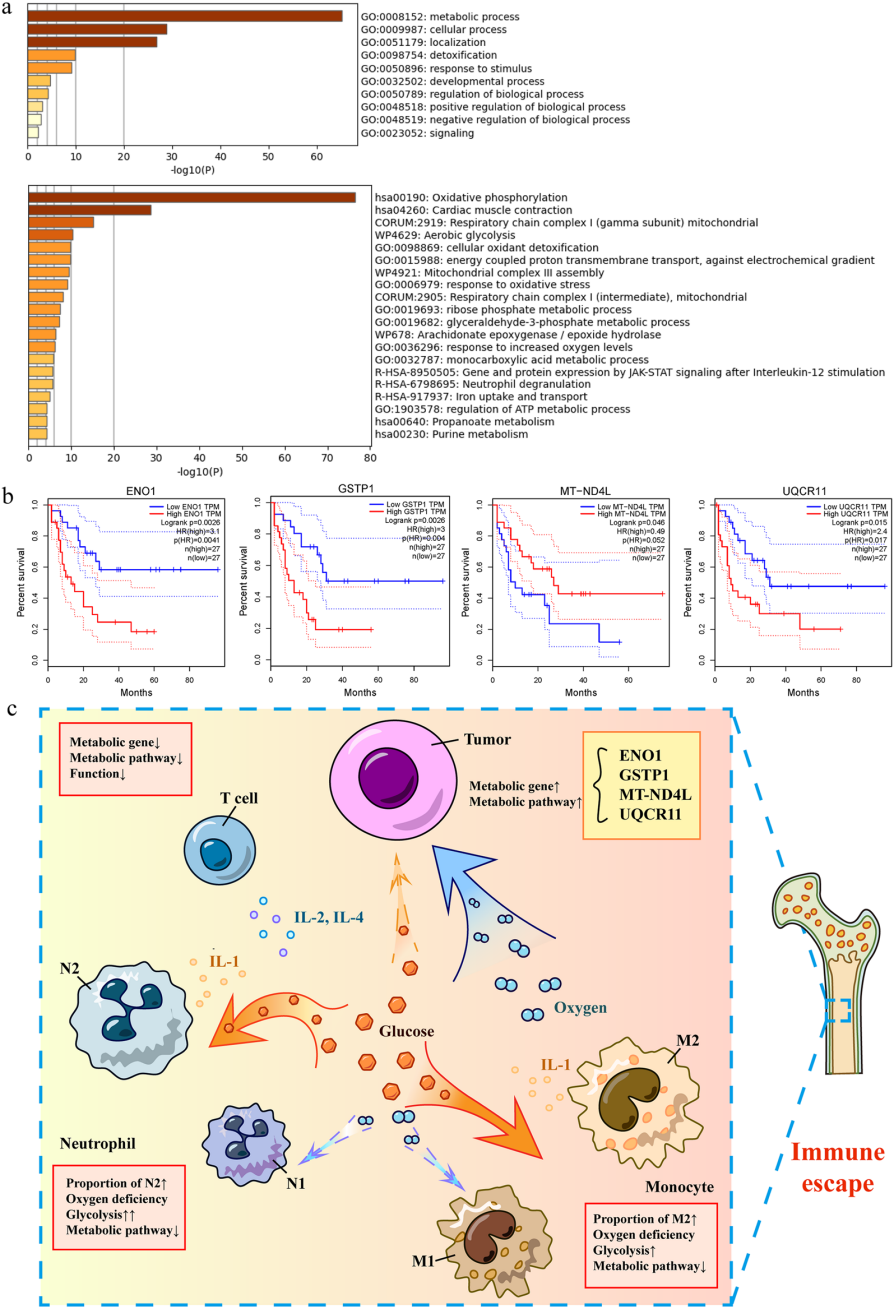
**a** Biological process and pathway enrichment. The top-scoring pathways related to metabolism and focused on OXPHOS, hinting at the metabolic characteristics of LSCs. **b** Survival analysis of AML metabolic differential genes (P < 0.005). **c** Immune escape mechanism of the AML bone marrow microenvironment. In the AML bone marrow microenvironment, leukemia cells compete with immune cells for nutrients and oxygen. Leukemia cells enhance their metabolic activity to satisfy energy demand. Myeloid immune cells are the major energy consumers and increase their proportions of anti-inflammatory cells, which have close communication with leukemia cells. Moreover, T cells weaken the cytotoxic effect by decreasing their metabolic gene expression levels and have little interaction with tumor cells. Furthermore, ENO1, GSTP1, MT-ND4L and UQCR11 are potential metabolic targets for AML treatment

## Conclusion

Overall, this study is the first to illustrate the immune evasion mechanism of leukemia cells from the perspective of metabolism, energy, oxygen and immunity of the bone marrow microenvironment by scRNA-seq. Crucially, potential metabolic targets of treatment with clinical significance were proposed (Fig. 2c), which is important for future studies to further explore metabolic treatments for AML. However, our work lacks AML samples for validation, and we need to verify these results in subsequent design experiments.

## Supplementary Information

**Additional file 1: Table S1.** The clinical characteristics of GSE130756. **Table S2.** The polarization and anti-inflammatory/pro-inflammatory gene sets of monocytes/neutrophils. **Table S3.** The relation between signaling pattern and metabolism in the TME. **Fig. S1.** Verification of the landscape of cell definition. **Fig. S2.** Heatmaps of gene expression level associated with key metabolic enzymes. **Fig. S3.** Polarization of myeloid immune cells in microenvironment. **Fig. S4.** GSEA analysis of insulin pathway and HIF pathway in AML progenitor cells and tumor-infiltrating immune cells. **Fig. S5.** The metabolic and oxygen preference of different monocyte subtypes in the TME. **Fig. S6.** The metabolic and oxygen preference of different neutrophil subtypes in the TME. **Fig. S7.** Cell**‒**cell communications in the TME. **Fig. S8.** Heatmaps of cytokines communication network in TME. **Fig.** **S9** Exploration of metabolic expression differential gene in AML progenitor cells.

## Data Availability

The datasets used and/or analysed during the current study are available in the GEO database.

## Abbreviations

AML: Acute myeloid leukemia
HSPCs: Hematopoietic stem/progenitor cells
GSVA: Gene set variation analysis
GSEA: Gene set enrichment analysis
TME: Tumor microenvironment
GO: Gene ontology
PPIs: Protein–protein interactions
