# Supplementary material for: The immunometabolic landscape of the bone marrow microenvironment in acute myeloid leukemia
Source: Exp Hematol Oncol. 2022 Oct 28;11:81. doi: 10.1186/s40164-022-00332-8 (PMC9615331; doi:10.1186/s40164-022-00332-8)
Supplement: Supplementary file 1 — Additional file 1: Table S1. The clinical characteristics of GSE130756. Table S2. The polarization and anti-inflammatory/pro-inflammatory gene sets of monocytes/neutrophils. Table S3. The relation between signaling pattern and metabolism in the TME. Fig. S1. Verification of the landscape of cell definition. Fig. S2. Heatmaps of gene expression level associated with key metabolic enzymes. Fig. S3. Polarization of myeloid immune cells in microenvironment. Fig. S4. GSEA analysis of insulin pathway and HIF pathway in AML progenitor cells and tumor-infiltrating immune cells. Fig. S5. The metabolic and oxygen preference of different monocyte subtypes in the TME. Fig. S6. The metabolic and oxygen preference of different neutrophil subtypes in the TME. Fig. S7. Cell‒cell communications in the TME. Fig. S8. Heatmaps of cytokines communication network in TME. Fig. S9 Exploration of metabolic expression differential gene in AML progenitor cells. [file 40164_2022_332_MOESM1_ESM.docx]

**Additional file 1**

**Methods:**

**Data availability and processing:**

The data are available at GEO (https://www.ncbi.nlm.nih.gov/gds/) under accession number GSE130756, and the files in the form of RAW.tar were downloaded. We used Seurat (https://satijalab.org/seurat/) in R 4.0 to process the data. For quality control, we filtered out cells in which fewer than 500 transcripts were detected. Then, we defined outlier cells according to the standard of mitochondrial ratio > 2-fold median absolute deviations, and cells with high proportions of mitochondria-encoded genes were also excluded. Then, the data were normalized via log2(TPM/100+1), and the percentage of mitochondria and the number of UMIs were regressed out. Additionally, we used reciprocal PCA (RPCA) and reference-based integration to harmonize the datasets. Subsequently, we performed a nonlinear dimensional reduction (t-SNE) analysis to show the clustering result. Then, we identified marker genes for each cluster using the function “FindAllMarkers”.

**Comparison analysis with the HCL:**

Because the AML data were too large to handle, we performed pseudo-cell processing of the AML landscape, with each pseudo-cell representing an average of 50 cells randomly selected from the same cell cluster. We used the R package “scHCL” with the average expression of features with the normalization data to help us identify cell types.

**Gene Set Variation Analysis:**

Gene set variation analysis (GSVA) is a nonparametric, unsupervised method for estimating the variation in gene set enrichment (https://github.com/rcastelo/GSVA). We downloaded KEGG gene sets as gene symbols (http://www.gsea-msigdb.org/gsea/downloads.jsp) and chose 63 metabolic pathways of interest. The normalized data were used to evaluate the enrichment score of each pathway with the “gsva” method, and we used “Gaussian” during the nonparametric estimation of the cumulative distribution function of expression levels across samples. Finally, we drew a heatmap to show the results.

**Gene Set Enrichment Analysis:**

Gene set enrichment analysis (GSEA) is a method to determine whether members of a gene set S tend to occur toward the top (or bottom) of the list L, in which case the gene set is correlated with the phenotypic class distinction. The gene sets were obtained from MsigDB, and we enriched the gene sets via fgsea (http://www.bioconductor.org/packages/release/bioc/html/fgsea.html).

**Cell‒cell communication:**

Cell‒cell communications were analyzed by CellPhoneDB and CellChat.

We installed CellPhoneDB in the environment with Python 3.8 and activated “cpdb-venv”. Then, the code was obtained from a website process (https://github.com/ventolab/CellphoneDB), and an intercellular communication heatmap was generated.

We loaded the CellChat package (https://github.com/sqjin/CellChat) and data in R, created the CellChat object, set the ligand‒receptor database as “CellChatdb.human”, and preprocessed the data. First, communication probability was calculated to infer the cellular communication network, and cell‒cell communication was inferred at the signal pathway level. Second, the aggregated cell‒cell communication network was calculated, and the weights between different networks were compared. Finally, each signal path was visualized using hierarchy, circle or chord diagrams. Additionally, we computed and visualized the network center fraction to display Senders (Sources) and Receivers (Targets) in two-dimensional space and identify the signals that contribute most to the incoming or outgoing signals in certain cell groups. To explore how multiple cell groups and signal paths coordinate functions, a pattern recognition method was adopted to identify global communication patterns.

**Differentially expressed genes:**

AML progenitor cells identified in “GSE92274” and HSPCs in “GSE130756” were randomly selected for differential expression gene (DGE) analysis using the “FindMarkers” function, and the "MAST" function was used for significance testing. Then, we set the following parameters: test. use = “MAST”, max-cells.per.ident = 1000, statistical significance was set as 0.0000001, and the difference multiple value was set as 1. In addition, when logFC was greater than 1, gene expression was regarded as upregulated, and when logFC was less than 1, gene expression was regarded as downregulated. Then, 478 differentially expressed genes were obtained. Next, we integrated metabolic pathways of interest in KEGG to obtain a total of 2,408 metabolic genes. Finally, 478 differential genes were intersected with 2,408 metabolic genes to obtain 69 metabolic differential genes, and ggplot2 was used to map the volcano plot.

**Survival analysis:**

Gene Expression Profiling Interactive Analysis (GEPIA: http://gepia.cancer-pku.cn/) is a TCGA data analysis and visualization website. We input the statistically significant metabolism-related differentially expressed genes in the navigation bar of the official website, and selected the "LAML" database in the “Survival” section. Finally, the survival curves were drawn.

**Metascape:**

Metascape (https://metascape.org/gp/index.html) is a powerful gene annotation tool that can help the user apply the current popular bioinformatics analysis method to the bulk analysis of genes and proteins. In this study, Gene Ontology (GO) analysis and Protein‒Protein Interactions (PPIs) analysis were performed via the Metascape website (https://metascape.org/gp/index.html).

**Table S1 The clinical characteristics of GSE130756.**

| **Sample ID** | **Age** | **Gender** | **FAB subtype** | **Therapeutic regimen** |
| --- | --- | --- | --- | --- |
| P01 | 19 | Female | M2 | NA |
| P02 | 66 | Male | M1 | NA |
| P03 | 40 | Male | M2 | IA |
| P04 | 56 | Female | M4 | IA, DAC+AAE, DAC+AAE |
| P05 | 52 | Male | M2 | IA, IA |
| P06 | 28 | Female | M5 | IA, HAE, CLAG, Ara-C+CLAG, AA |
| P07 | 53 | Male | M0 | IA, IA |
| P08 | 71 | Female | M0 | DAC+IA, IA, IA |
| P09 | 18 | Female | M2 | IA, IA, IDA+Ara-C, VP-16+Ara-C |
| P10 | 29 | Male | M2 | IA, IA, Ara-C, Ara-C, DAC+IA, DAC+IA |
| P11 | 35 | Female | M2 | IA,CAG,DAC+CAG, DAC+AA, Ara-C, Ara-C, Ara-C, HAE |
| P12 | 64 | Female | M2 | NA |
| P13 | 68 | Female | M2 | NA |
| P14 | 67 | Female | M5 | DAC+IA, AZA+AA, AZA+AA, AZA+AA |
| P15 | 63 | Male | M5 | IA, IA |
| P16 | 24 | Female | M5 | IA, CAG, CALG |
| P17 | 50 | Male | M5 | IA, IA, IA, IA, IA |
| P18 | 53 | Male | M2 | IA, IA |
| P19 | 28 | Male | M4 | IA, IA, AAE |
| P20 | 29 | Male | M5 | IA, IA, FLAG, AAE, VP-16+Ara-C, MEA, MEA |
| P21 | 53 | Male | M4 | IA, CAG |
| P22 | 67 | Female | M2 | DAC+CAG |
| P23 | 38 | Female | M5 | IA, IA, IA, EA, Ara-C |
| P24 | 43 | Male | M2 | IA, IA, Ara-C, IA, AAE |
| P25 | 56 | Female | M5 | IA, IA, IA, Ara-C, Ara-C, Ara-C |
| P26 | 18 | Male | M2 | IA, IA, HAA |
| P27 | 49 | Female | M5 | IA, IA, IA, AAE, AAE, AAE |
| P28 | 65 | Male | M2 | NA |
| P29 | 65 | Male | M1 | DAC+CAG |
| P30 | 57 | Male | M5 | DAC+CAG, DAC+CAG, DAC+CAG, DAC+CAG |
| P31 | 39 | Male | M5 | IA, IA, Ara-C, HAA, HAA |
| P32 | 40 | Male | M5 | IA, IA, IA, Ara-C, Ara-C, Ara-C, Ara-C |
| P33 | 63 | Male | M4 | DAC+CAG, DAC+CAG, AZA+IA |
| P34 | 62 | Male | M2 | DAC+CAG, DAC+IA, DAC+IA, DAC+IA |
| P35 | 24 | Female | M5 | IA, IA, Ara-C, Ara-C, Ara-C, IA |
| P36 | 61 | Male | M5 | DA, IA, AAE, AAE, HAA |
| P37 | 66 | Female | M5 | DAC+IA |
| P38 | 71 | Male | M2 | DAC+IA |
| P39 | 29 | Male | M2 | IA, IA, Ara-C, Ara-C, AAE |
| P40 | 70 | Male | M2 | IA, IA, IA, IA, IA |
| N01 | 60 | Female | Normal |  |
| N02 | 63 | Male | Normal |  |
| N03 | 44 | Female | Normal |  |

**Table S2 The polarization and anti-inflammatory/pro-inflammatory gene sets of monocytes/neutrophils.**

|  | Marker gene |
| --- | --- |
| M1/N1 Polarization | IL12, IL23, IL12, TNF, IL6, CD86, IL1B, MARCO, NOS2, IL12. FCGR1A, CD80, CXCR10, IL23, CXCL9, CXCL10, CXCL11, CD86, IL1A, IL1B, IL6, TNF, CCL5, IRF5, IRF1, CD40, IDO1, KYNU, CCR7 |
| M2/N2 Polarization | ARG1, ARG2, IL10, FCGR2A, CD163, FCER2, CD200R1, PDCD1LG2, CD274, MARCO, CSF1R, MRC1, IL1RN, IL1R2, IL4R, CCL4, CCL13, CCL20, CCL17, CCL18, CCL22, CCL24, LYVE1, VEGFA, VEGFB, VEGFC, VEGFD, EGF, CTSA, CTSB, CSTC, CTSD, TGFB1, TGFB2, TGFB3, MMP14, MMP19, MMP9, CLEC7A, WNT7B, FASLG, TNFSF12, TNFSF8, CD276, VTCN1, MSR1, FN1, IRF4 |
| Anti-inflammatory | IL1RN, IL10, IL4, IL11, IL13, TGFB1, TNFRSF1A, TNFRSF1B, IL1R2, IL18BP |
| Pro-inflammatory | IL1B, TNF, CCL2, CCL3, CCL5, CCL7, CCL8, CCL13, CCL17, CCL22 |

**Table S3 The relation between signaling patterns and metabolism in the TME.**

| **Signal** | **metabolism** | **PMID** |
| --- | --- | --- |
| **BMP** | Regulates the formation and function of energy metabolism, especially glucose and fatty acid metabolism | 32512164 |
| **ACTIVIN** | Regulates glucose/energy metabolism via promoting the differentiation of insulin-producing and responsive cells, and regulating the function of differentiated cells | 21353883 |
| **NRG** | Induces glucose uptake and regulates the glucose metabolism of cardiac protein | 33063822 |
| **FGF** | Regulates the expression of glycolytic enzyme hexokinase 2 by FGF-dependent control of c-MYC, supporting glycolysis | 28467822 |
| **VEGF** | Increases the expression of GLUT-1 via activating PI3K-AKT signaling pathway in endothelial cells, which promotes the uptake and transport of glucose; promotes lipid uptake and transport to peripheral tissues by upregulating the expression of FATP3 and FATP4 | 23973331 |
| **IGF** | Is related to glucose metabolism and lipid metabolism; lowers circulating amino acids by reducing protein breakdown | 26733412  2651479 |
| **IL-2** | Promotes glycolysis, oxidative phosphorylation, and synthetic processes, such as biosynthesis of protein, lipid and nucleotide biosynthesis via mTOR signal | 30619342 |
| **IL-4** | Enhances the phosphorylation of AKT and GSK-3α/β, thereby promoting glycogen synthesis; promotes the energy storage by increasing insulin-stimulated glucose uptake and lipid synthesis in liver | 30584465 |
| **OSM** | Upregulates pentose and glucoronate interconversion, ascorbate and aldarate metabolism | 34124061 |
| **IL-1** | Plays an important role in lipid metabolism through regulating insulin level and lipase activity under physiological conditions; potentiates the action of glucagon on hepatic amino acid uptake and utilization | 12975454  8028504 |
| **PRL** | Plays an important role in glycolytic metabolism by increasing IL-8 secretion in colorectal cancer cells | 28791350 |
| **TRAIL** | Promotes the uptake of glucose by TRAIL/DR5 signaling pathway | 34789726 |
| **BAFF** | Regulates the metabolism of B cell, promoting its function | 34174517 |
| **RESISTIN** | Decreases glucose uptake, glycolysis rate and ATP production; reduces the uptake and metabolism of fatty acids | 30076527  16137686 |
| **COMPLEMENT** | Regulates the metabolism of T cells | 32166778 |


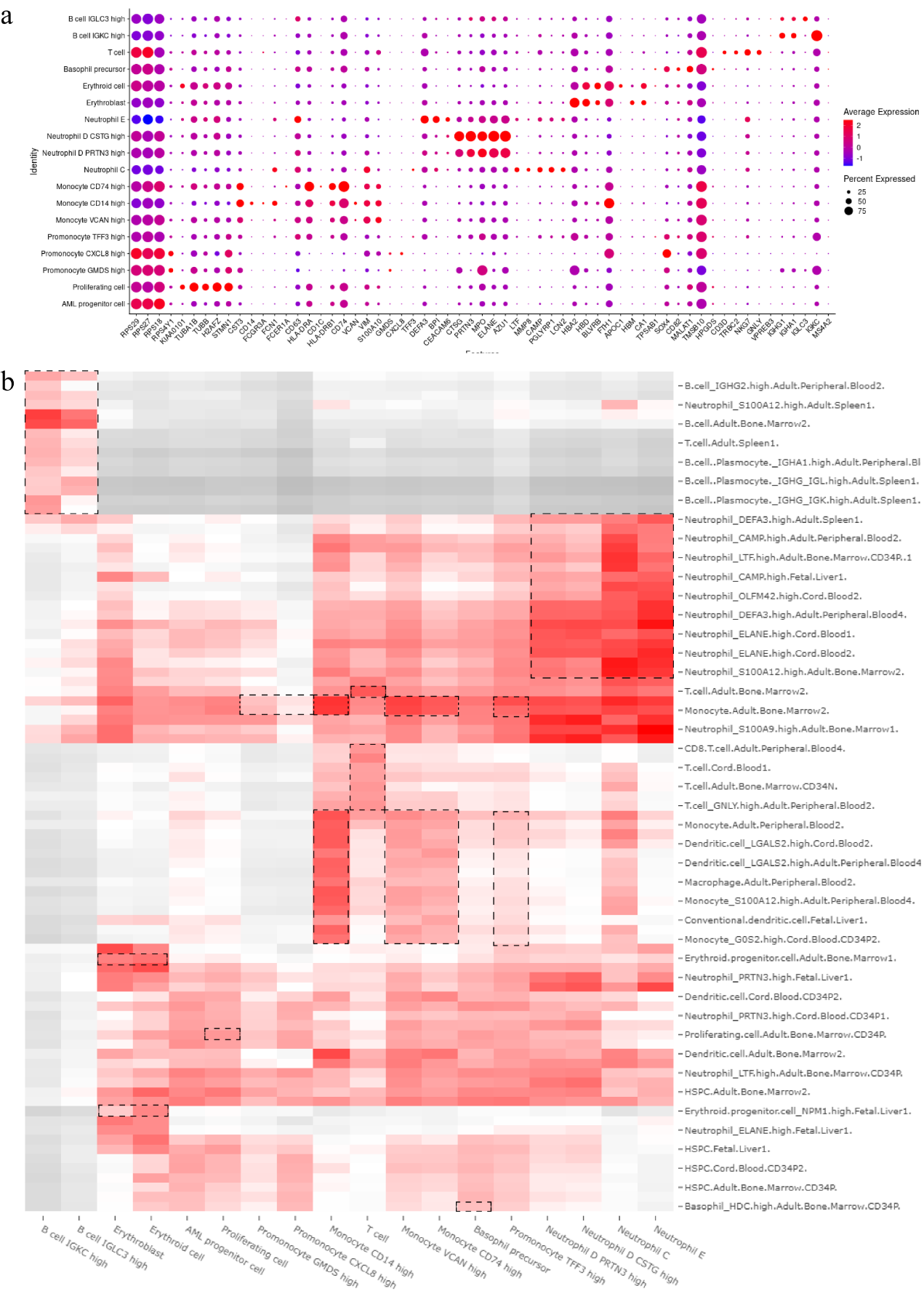


**Fig. S1 Verification of the cell definition.** a Bubble diagram of marker genes. b HCL mapping heatmap. The cell definition is confirmed by single-cell mapping pipeline basing on the HCL database.


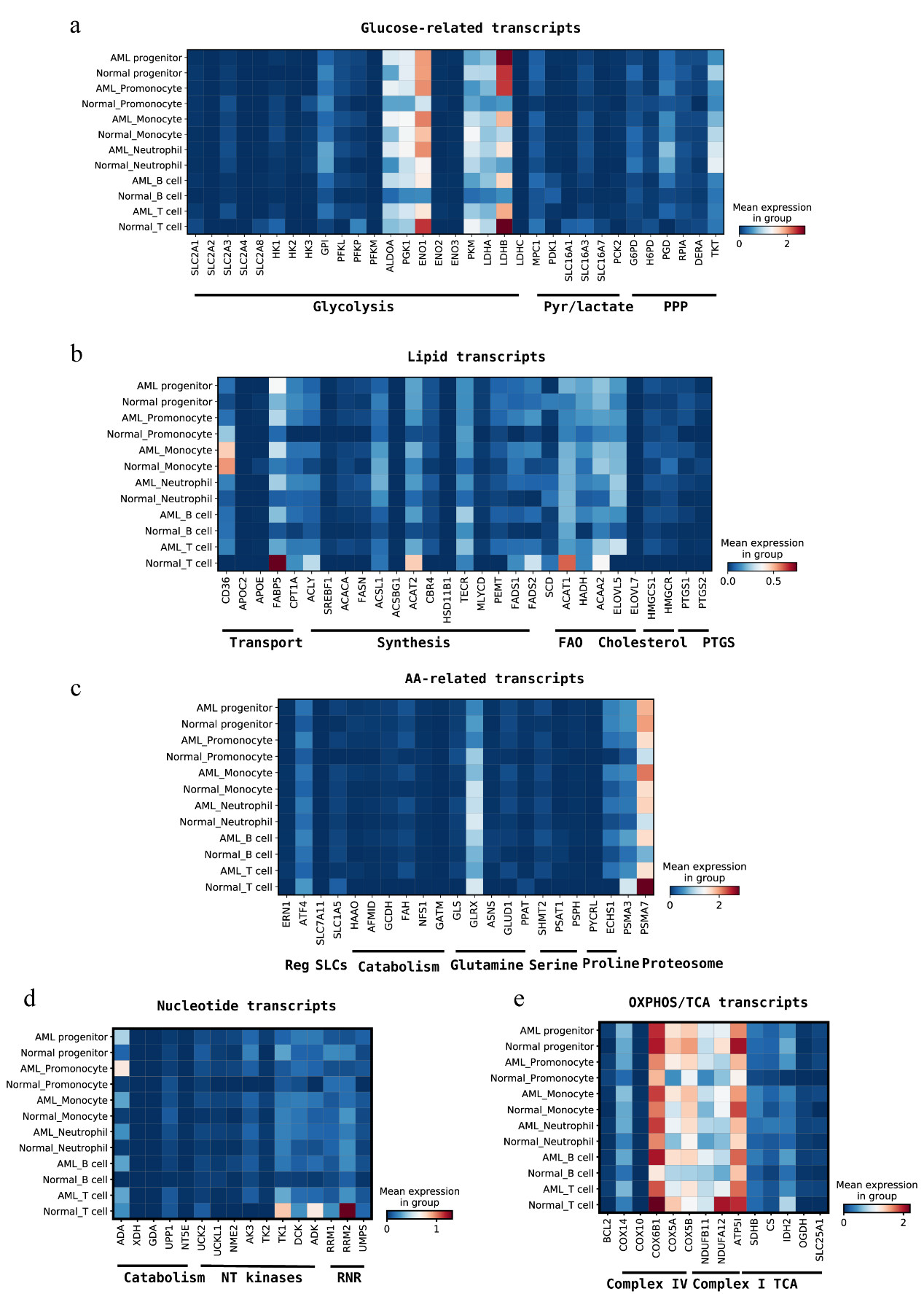


**Fig. S2 Heatmaps of gene expression level associated with key metabolic enzymes.** a-e **Heatmaps** of glucose-related, lipid, amino acid (AA)-related, nucleotide and OXPHOS/TCA transcripts. The expression of metabolic enzymes gene increased in the TME except for T cells.
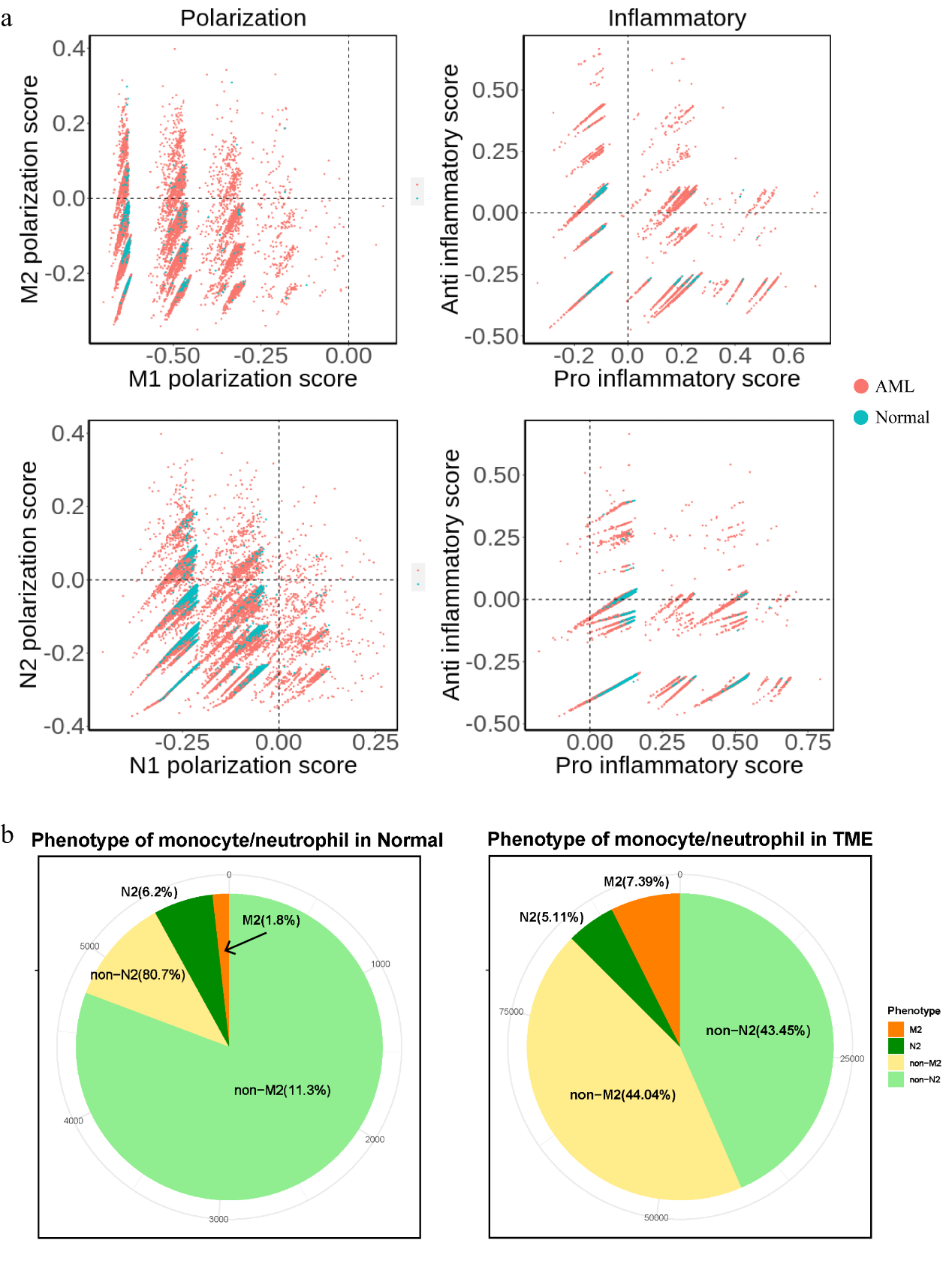


**Fig. S3** **Polarization of myeloid immune cells in microenvironment.** a Polarization/inflammatory score of neutrophils and monocytes; b Polarization of monocytes/neutrophils in the normal and AML bone marrow microenvironment. The proportion of neutrophils and monocytes polarized toward anti-inflammatory phenotype in TME is more than in the normal microenvironment.


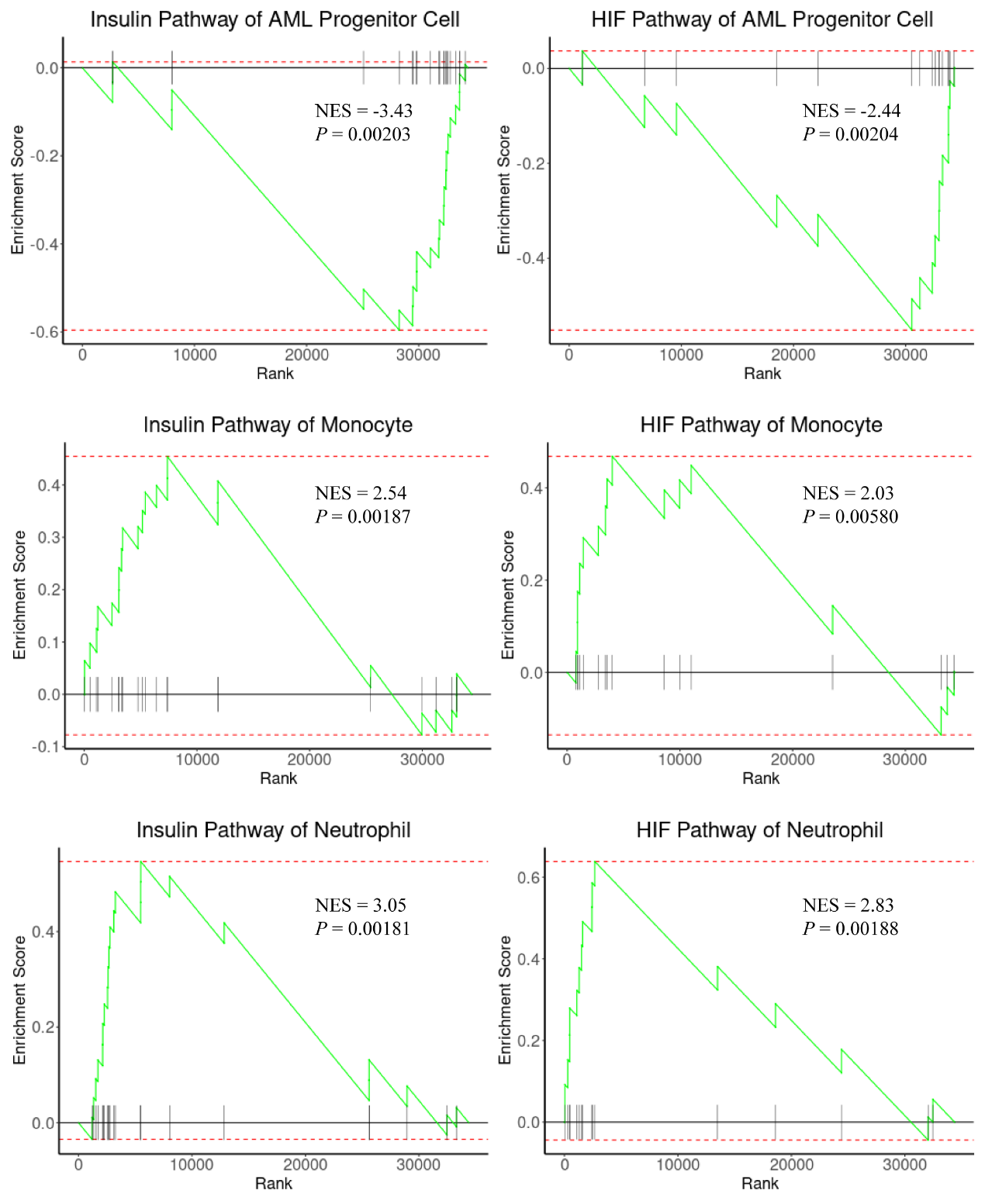


**Fig. S4 GSEA analysis of insulin pathway and HIF pathway in AML progenitor cells and tumor-infiltrating immune cells.** Insulin pathway and HIF pathway are enriched in tumor infiltrating neutrophils and monocytes but not in AML progenitor cells.
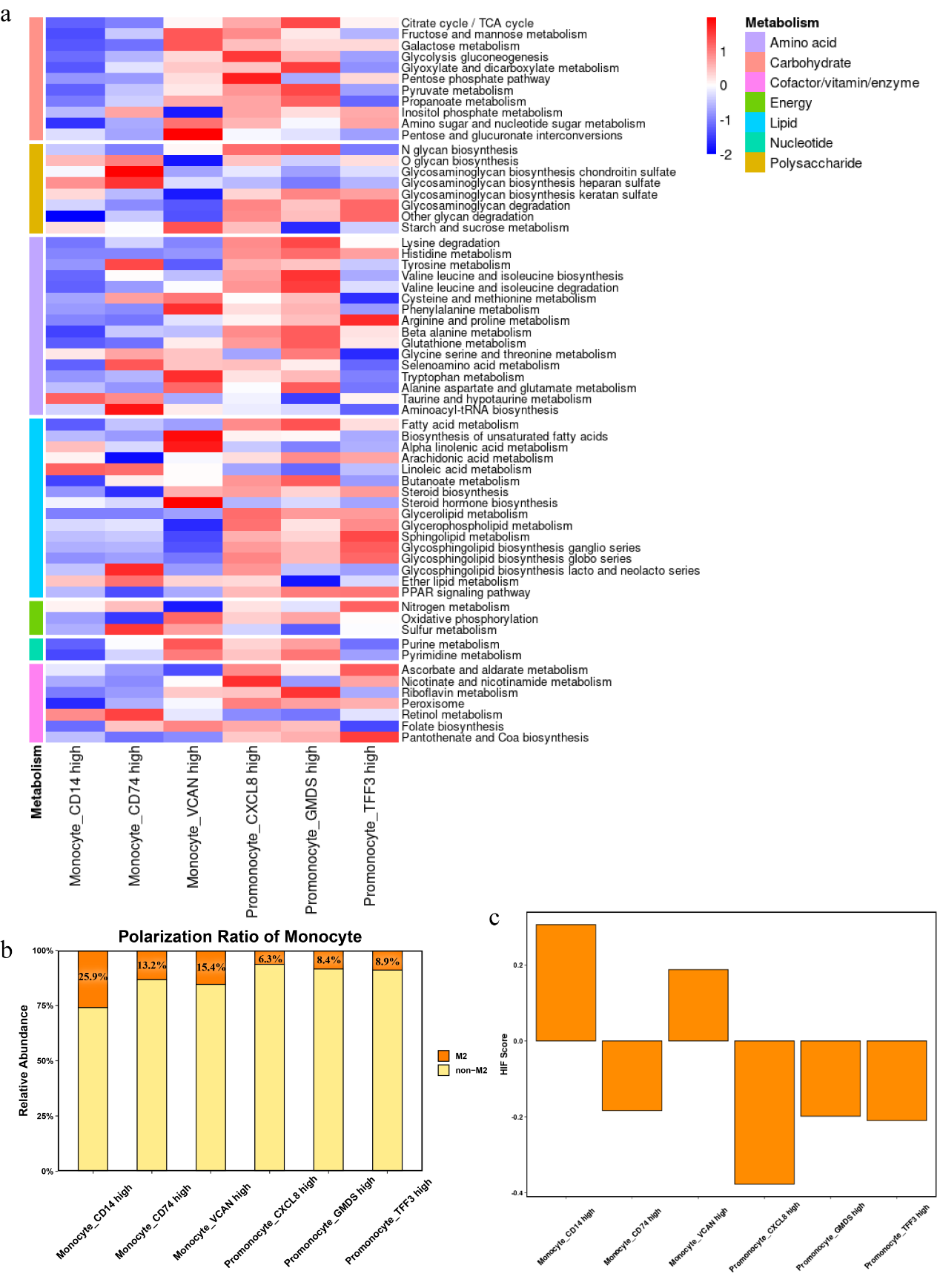


**Fig. S5 The metabolic and oxygen preference of different monocyte subtypes in the TME.** a GSVA analysis of metabolic pathways of different subtypes of monocytes. b The polarization ratios of different subtypes of monocytes. The populations of tumor-derived monocytes with active metabolism have lower proportions of anti-inflammatory phenotype. c HIF pathway scores in different subtypes of monocytes. The more hypoxia the monocytes were, the less metabolically active they were.

**
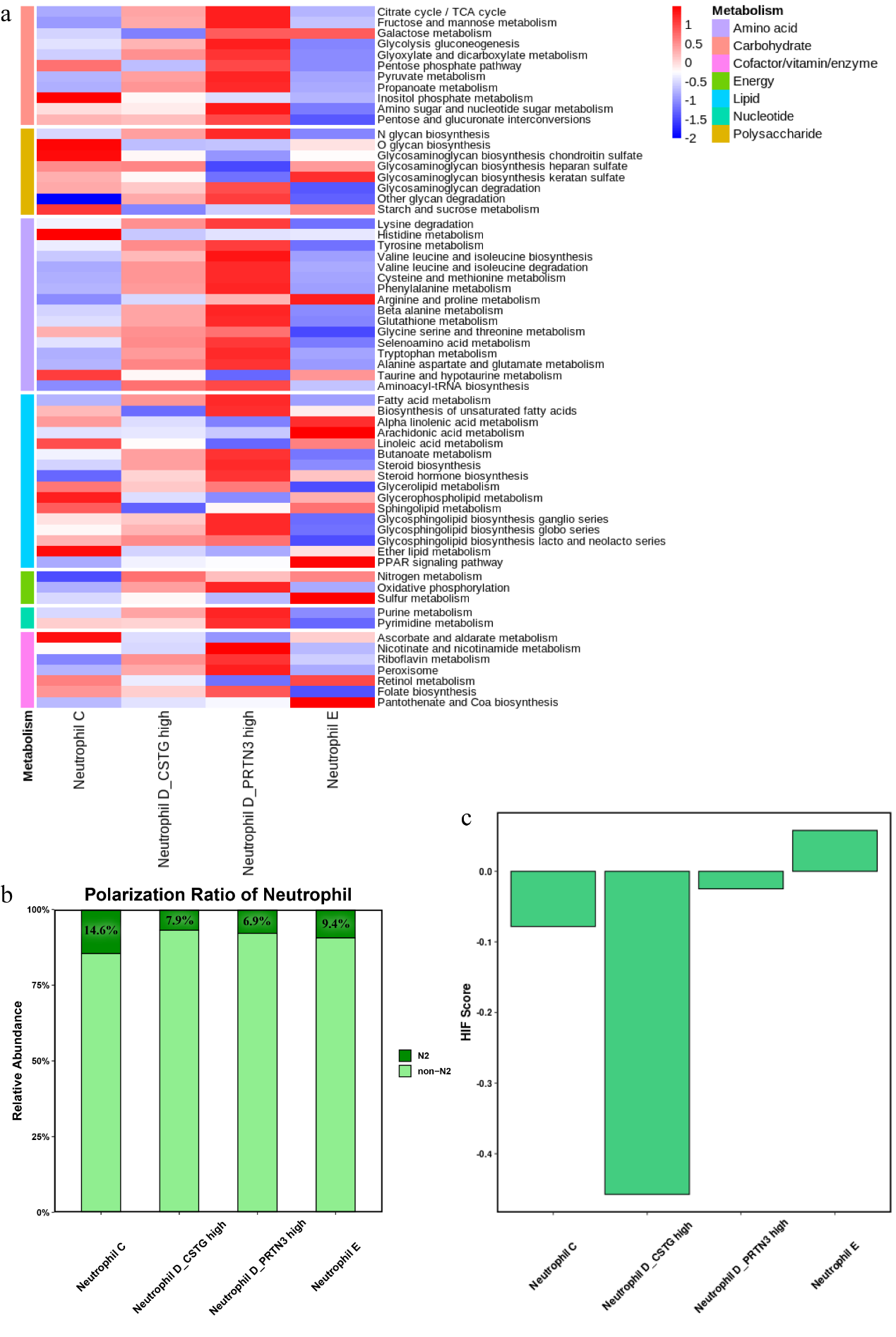
**

**Fig. S6 The metabolic and oxygen preference of different neutrophil subtypes in the TME.** a GSVA analysis of metabolic pathways of different subtypes of neutrophils. b The polarization ratios of different subtypes of neutrophils. The populations of tumor-derived neutrophils with active metabolism have a lower proportion of anti-inflammatory phenotype. c HIF pathway scores in different subtypes of neutrophils. The more hypoxia the neutrophil were, the less metabolically active they were.


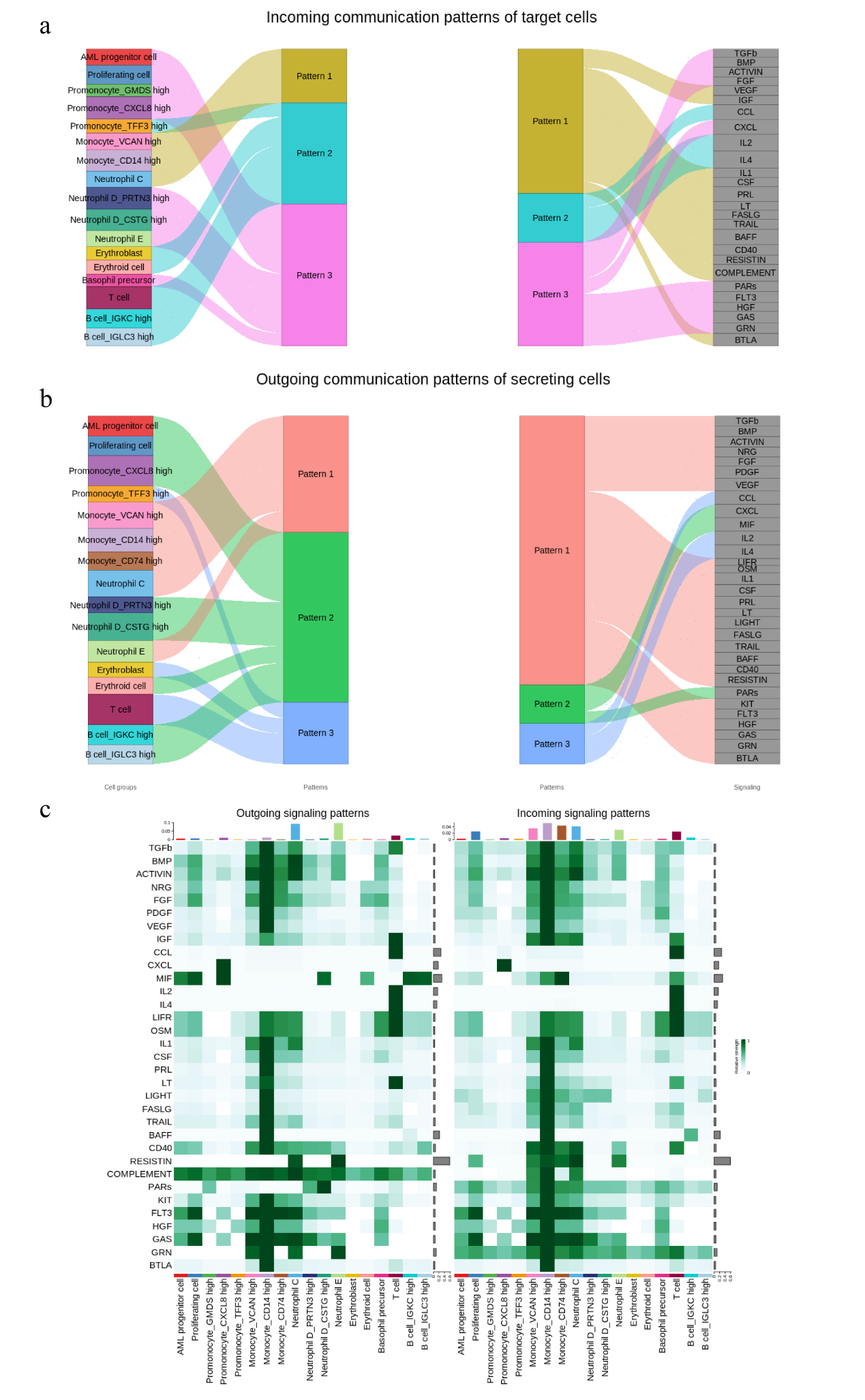


**Fig. S7 Cell‒cell communications in the TME.** a The incoming communication patterns of target cells. b The outgoing communication patterns of secreting cells. c The heatmap of outgoing/incoming signal patterns in the AML bone marrow microenvironment.


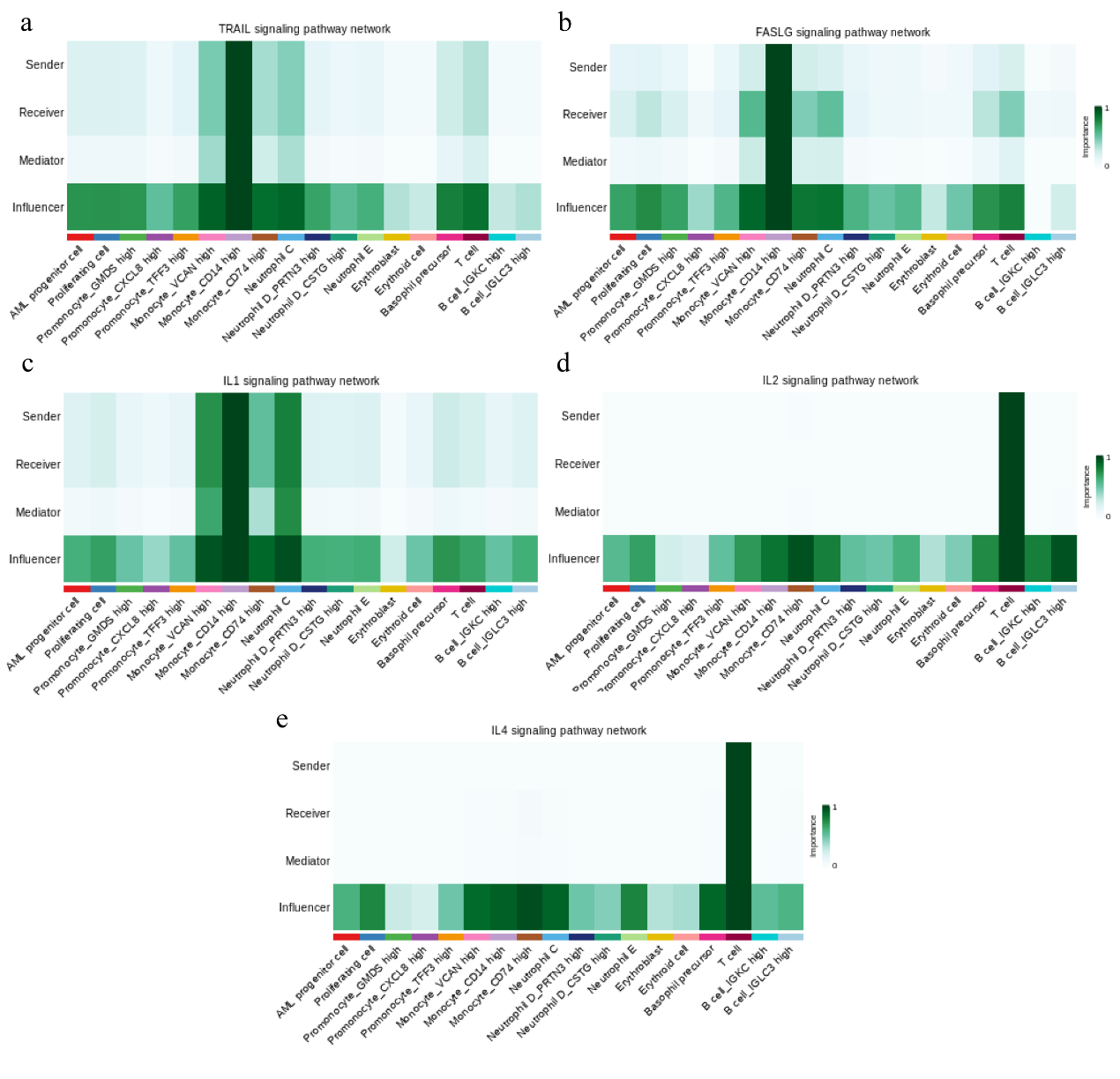


**Fig. S8 Heatmaps of the cytokines communication network in the TME.** a-e Signaling pathways of TRAIL, and FASLG, IL-1, IL-2, and IL-4 in TME. The monocyte_CD14 high is the main sender, receiver, mediator and influencer of TRAL and FASLG signals. Notably, T cells are mainly composed of IL-2 and IL-4 signals, involving in the sending, receiving, mediating and influencing of such signals, while IL-1 is mainly involved in the communication of monocytes and neutrophils. In addition, IL-1, IL-2 and IL-4 have an action on AML progenitor cells.


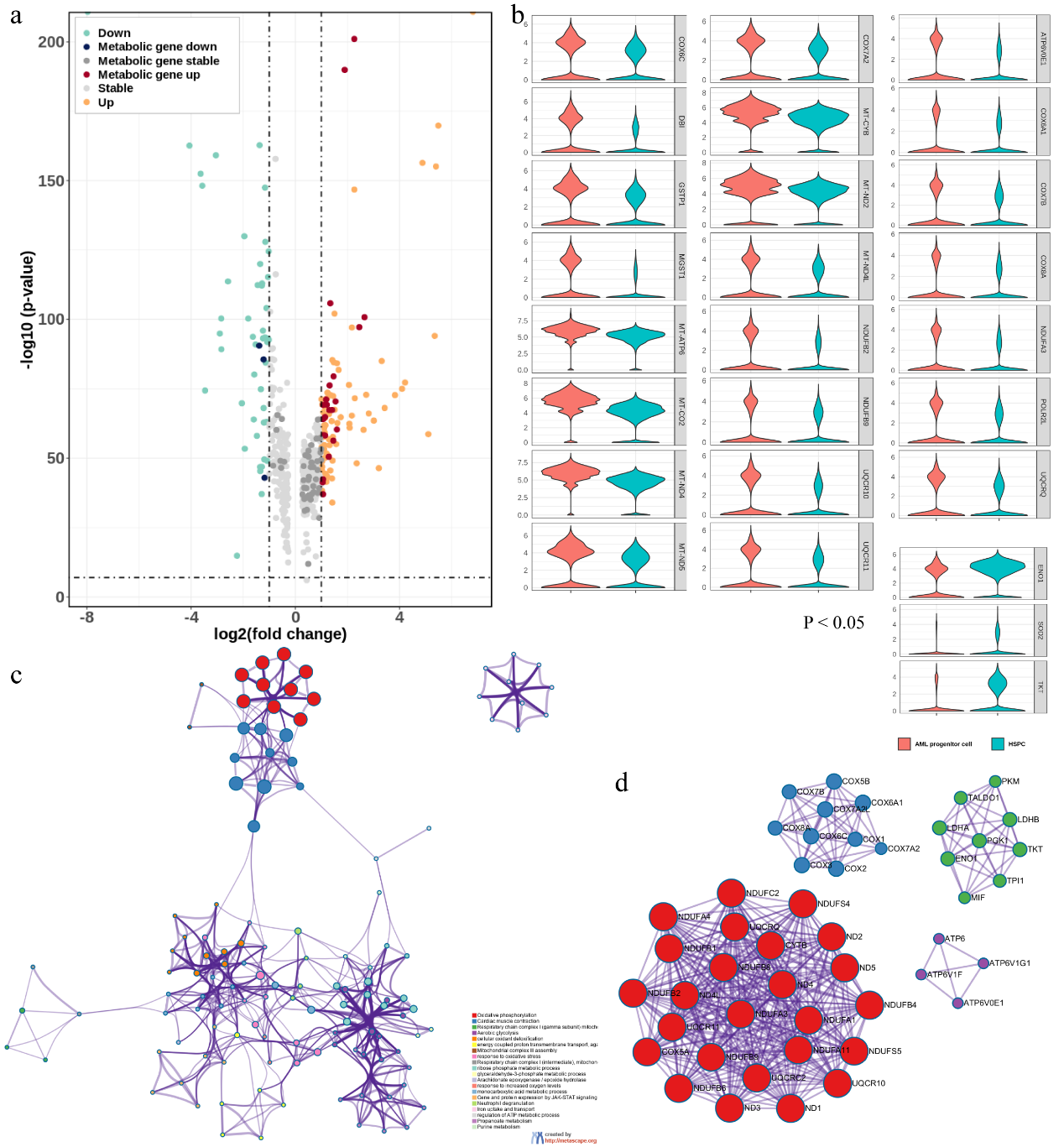


**Fig. S9 Exploration of metabolic differential expression gene in AML progenitor cells.** a The volcano map of metabolic differential expression genes related to AML progenitor cells and HSPCs (*P* < 0.0000001); b Violin plots of upregulated and downregulated metabolic gene (*P* < 0.05). c-d Two patterns of manifestation of PPIs networks of metabolic differential genes. The protein related to metabolic differential expression gene of AML progenitor cells can be divided into 4 modules, associating with NADH dehydrogenase, cytochrome oxidase, ATP hydrolytic, proton transmembrane transport activity and key enzymes of glucose metabolism respectively.
